# Using the Horseshoe Crab, *Limulus Polyphemus*, in Vision Research

**DOI:** 10.3791/1384

**Published:** 2009-07-03

**Authors:** Jiahui S. Liu, Christopher L. Passaglia

**Affiliations:** Department of Biomedical Engineering, Boston University

## Abstract

The American horseshoe crab, *Limulus Polyphemus* is one of the oldest creatures on earth, and the animal continues to play an indispensable role in biomedical research. Not only does their blood contain special cells that scientists use to detect bacteriotoxins in our medicines, but their eyes also contain a neural network that has provided much insight about physiological processes operating in our visual system, such as light adaptation and lateral inhibition. The horseshoe crab remains an attractive model for vision research because the animal is large and hardy for an invertebrate, its retinal neurons are big and easily accessible, its visual system is compact and extensively studied, and its visual behavior is well defined. Moreover, the structure and function of the eyes are modulated on a daily basis by a circadian clock in the animal s brain. In short, the visual system of horseshoe crabs is simple enough to be understood yet complex enough to be interesting.

In this video we present three electrophysiological paradigms for investigating the neural basis of vision that can be performed in vivo with Limulus. They are electroretinogram recording, optic nerve recording, and intraretinal recording. Electroretinogram (ERG) recordings measure with a surface electrode the summed electrical response of all cells in the eye to a flash of light. They can be used to monitor the overall sensitivity of the eye for prolong periods of time. Optic nerve recordings measure the spiking activity of single nerve fibers with an extracellular microsuction electrode. They can be used to study visual messages conveyed from the eye to the brain as well as circadian-clock messages fed back from the brain to the eye. Intraretinal recordings measure with an intracellular microelectrode the voltage fluctuations induced by light in individual cells of the eye. They can be used to elucidate cellular mechanisms of retinal processing.

**Figure:**
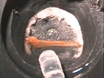


## Protocol

### Part 1: Experimental Preparation

Experimental procedures performed on horseshoe crabs have been approved by the Institutional Animal Care and Use Committee at Boston University. Animals are purchased from the Marine Biological Labs (Woods Hole, MA) or other vendor and housed in an aerated saltwater tank in a room exposed to a regulated light-dark cycle. The lighting regimen is important for entraining the crab's circadian clock and cycling the eye daily between its daytime and nighttime states. Immediately before starting any invasive procedures, the animal is chilled in an ice bucket for 10-15 minutes until its motion is slowed and then secured to a wooden platform with two stainless steel screws inserted in the prosoma and two in the opisthosoma. The platform is weighted underneath with granite so that it sinks in water. After terminal experiments the animal is euthanized by immersing into ice slurry again and pithing the brain, which lies above the mouth, with a scalpel.

### Part 2: Solutions

Limulus Ringer's solution is composed of 430 mM NaCl, 9.56 mM KCl, 9.52 mM CaCl2, 9.97 mM MgCl2, 21.05 mM MgSO4, 50μM TES, 50μM HEPES, and 10ml/L Pen-Strep mix (Penicillin-Streptomycin, 10000 units/ml).

### Part 3: Electroretinogram Recording

Tools needed for this procedure include a screwdriver, petroleum jelly, Ringer's solution, a transfer pipette, a green LED, a recording chamber, stainless steel screws, and a cotton swab.

A handmade chamber is used to record the ERG (Figure 1A). The chamber body is designed to hold a saline reservoir in contact with the eye. The lid contains a silver chloride wire for coupling the conductive solution to an amplifier and a small hole sized to accommodate an LED. An ultrabright LED with peak emission around 520nm works best.
To start, the underside of the chamber is coated with petroleum jelly and secured over the eye with two screws.
The chamber is then filled with saline and capped with the lid.
After chamber attachment the crab is placed in a light-tight cage in a tank filled with seawater over the gills.
The LED cable is inserted into the chamber lid.
The signal lead is clipped to the chloride wire, and the reference lead is clipped to one of the implanted screws.
Both leads are connected to the head stage of a high-impedance differential amplifier (X-cell 3x4, FHC Inc) for signal amplification and the amplifier filter is set to pass frequencies below 10Hz.
The amplifier output is fed to an oscilloscope for viewing and to a data acquisition board for computer analysis and storage.
After setup the cage door is closed to block room light from reaching the animal.
Data are collected with a custom program written in LabView. The program flashes the LED, records the evoked ERG signal, and measures the peak-to-peak response amplitude. The choice of flash paradigm depends on the experimental objective. For example, a brief (100ms) pulse of 5V applied every 10 minutes to the LED avoids effects of light adaptation when monitoring circadian changes in eye sensitivity.

### Part 4: Optic nerve recording

Tools needed for this procedure include a screwdriver, thread, a trephine, Ringer's solution, rongeurs, vannas scissors, a recording chamber, curved forceps, fine needle probes, a dull scalpel, surgical scissors, and a suction electrode.

A handmade chamber is used to record optic nerve responses (Figure 1B). The interior of the chamber is 2cm-diameter open well that has a thin semicircular slotted opening in the bottom wall to accommodate the nerve and isolate it from surrounding tissue.
Before chamber attachment a 16-gauge needle is inserted between the hinge muscles into the heart and ~20cc of blood is drained from the animal. Exsanguination is not necessary but makes optic nerve dissection easier.
The location of the optic nerve is estimated by drawing on the carapace a slightly curved line between the lateral and median eyes.
A circular hole is then cut in the carapace with a trephine. The hole is the same diameter as the chamber well. The center of the hole is located 2-3 cm anterior to the lateral eye and slightly dorsal to the line so that the nerve runs along the ventral portion of the well.
Connective tissue is cleared until the nerve is fully visible and free from surrounding and underlying tissue.
A strand of thread is looped around the nerve and pulled into the chamber through the slot at the bottom. Through this same opening the nerve is gently guided into the chamber by pulling on the string. At the same time the chamber well is inserted into the hole. The chamber is then affixed to the carapace with screws and the well is filled with Ringer's solution.
After chamber attachment the animal is placed in a light-tight cage in a tank filled with seawater over the gills.
The well is visualized under a stereoscope (SMZ-168, Jed Pella Inc) and cotton is padded around the opening in the bottom to prevent leakage of blood into and saline out of the chamber.
Residual connective tissue around the nerve is removed with fine scissors and tweezers, and the chamber is refilled with fresh Ringer's solution.
A nick is made in the blood vessel that encapsulates the nerve and through this opening the nerve is carefully desheathed along its length using fine scissors, tweezers, and needle probes.
A tiny fiber bundle is separated from the nerve using the probe and cut at the end furthest from the eye for afferent fiber recording and at the end closest to the eye for efferent fiber recording. In the video the end for afferent fiber recordings is cut.
A microsuction electrode (A-M Systems Inc) filled with Ringers solution is used for optic nerve recording. The electrode tip is fitted to the nerve bundle by fire polishing the end of a 1mm diameter borosilicate glass capillary.
The electrode tip is lowered into the chamber well with a manual manipulator (WPI Inc), and the cut fiber bundle is sucked into the tip. Suction is provided by a Gilmont syringe connected to the electrode via tubing.
A BNC connection provides the signal lead and a silver chloride wire wrapped around the electrode to reduce noise provides the reference lead. The two leads are connected to the head stage of a differential amplifier for signal amplification and noise filtering (X-cell 3x4, FHC Inc). The amplifier output is sent to an oscilloscope for viewing and a data acquisition card for computer analysis and storage.
Data are collected with a custom program written in LabView. The program controls light stimuli via a digital video processor (Bits++, Cambridge Research Systems Inc) and interfaces with a digital spike discriminator (APM, FHC Inc) that records spike trains. Light can be delivered to single cells in the eye via a fiber optic light pipe or to the entire eye via a computer-controlled video display.

### Part 5: Intraretinal recording

Tools needed for this procedure include a screwdriver, a L-shaped lucite platform with threaded screw holes, a microelectrode holder with a glass pipette, stainless steel screws, tweezers, and a fine scalpel.

A handmade lucite plate with preset screw holes is used to record intracellular responses. The holes are spaced for mounting a motorized micromanipulator (PPM5000, WPI Inc). The plate is attached to the animal with two screws on the side and one on top.
After plate attachment the animal is placed in a light-tight cage in a tank filled with seawater over the gills.
The micropositioner is screwed into the plate, with its movable arm aligned over the eye.
A batch of glass micropipettes are pulled from 1-mm OD borosilicate glass and the tips are backfilled via capillary action by placing the micropipettes in a small vial of 3M KCl solution.
The rest of the pipettes are then filled manually with the salt solution and inserted into an electrode holder.
The electrode holder is connected to the head stage of an intracellular amplifier (IR-283, Cygnus Technology Inc) affixed to the micropositioner arm.
A tiny section of retina (~1mm2) is exposed by cutting away the corneal interface with a razor blade.
A drop of Ringer's solution is placed on the exposed retina to prevent drying and the micropipette tip is advanced through the opening into retinal tissue.
When the tip enters the solution, the current injection mode of the intracellular amplifier is engaged and electrode impedance is measured. Micropipettes with impedance outside the range of 20-70 MW are discarded. Those in this range are advanced in micron steps and impaled into cells by vibrating the pipette tip electronically or mechanically.
Three types of cells may be encountered in the eye. Retinular cells show only a depolarizing response to light, eccentric cells show a train of action potentials riding on a depolarizing response, and pigment cells show no light response at all.
Data are collected with a custom program written in Labview. The program controls light stimuli via a digital video processor (Bits++, Cambridge Research Systems). The stimuli are delivered to impaled cells with a fiber optic pipe or video display. Voltage responses are observed on the oscilloscope and digitized by the program to computer.

### Part 6: Representative Results

A representative ERG is shown in Figure 2A. The waveform represents the summed electrical response to light of primarily the photoreceptive retinular cells, as they greatly outnumber the eccentric cells and are electrically coupled to them. There are not multiple waveform components from different retinal cell types as with the mammalian ERG. Circadian feedback from a clock in the Limulus brain modulates the physiological properties of retinal cells on a daily basis, causing the amplitude and time course of the ERG to vary over time (1). As shown in Figure 2B, ERG amplitude is highest during the animal's subjective night and lowest during the subjective day.

A representative trace of a single optic nerve fiber response to light is shown in Figure 3. Eccentric cells all behave roughly the same, showing a transient increase in spike discharge rate followed by a decay to a sustained level. The rate decay reflects the combined action of light adaptation and spike-dependent self-inhibition on eccentric cells (2). Other spike patterns, such as light-dependent decreases in rate, are seen in the Limulus brain but not the eye (3).

Representative traces of the voltage response of a pigment cell, retinular cell, and eccentric cell to light are shown in Figure 4. Only the latter two retinal cell types are visual. The amplitude and time course of their response depends on the quality of the recording and the location of the electrode within the cell. Usually, the electrode penetrates pigment cells or retinular cells because of their large size and number. If the latter, a transient depolarization that decays to a sustained level is recorded. The decay is due to light adaptation by retinular cells (2). If the electrode enters an eccentric cell, large action potentials are recorded in the axon (40-70mV) and small action potentials riding on a depolarizing waveform (<25mV) near the soma.


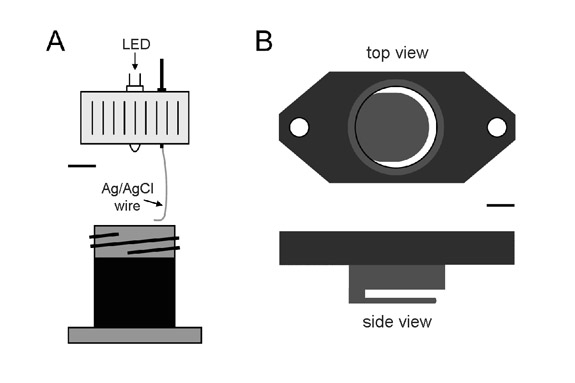
 **Figure 1.** Schematic diagrams of chambers used for electroretinogram recording (A) and optic nerve recording (B). Bar equals 7mm in A and 5mm in B.


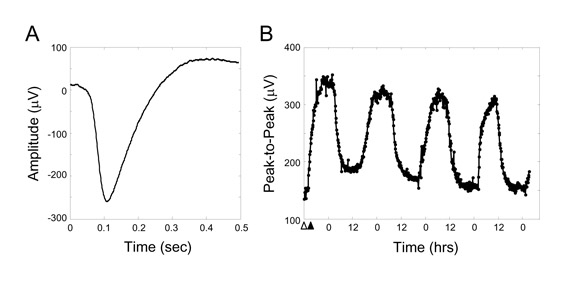
 **Figure 2.** Example trace of a Limulus ERG evoked by a 100ms LED pulse of 5V in the dark (A) and the peak-to-peak fluctuation in ERG amplitude over time in constant darkness (B). Darkness was initiated at the time indicated by the white triangle. The growth in ERG amplitude through the time indicated by the black triangle is due to light adaptation, which increases eye sensitivity in the dark. The cyclic variation in ERG amplitude thereafter is due to the animal's internal circadian clock. Time points in B are every 5min.


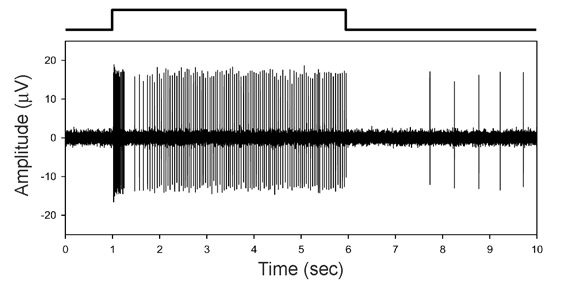
 **Figure 3.** Example trace of an optic nerve fiber response to light flashed on a single ommatidial receptor. Waveform above the trace shows stimulus timing. Eccentric cells, whose axons give rise to nerve fibers, all show a similar pattern of firing as this under the illumination conditions of the experiment (5sec flash in total darkness). Retinal coding with spikes is a property Limulus has in common with mammals, unlike other invertebrates.


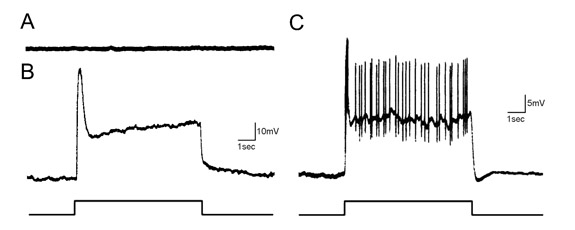
 **Figure 4.** Example traces of the voltage response to light of the three cell types present in the Limulus lateral eye: pigment cell (A), retinular cell (B), and eccentric cell (C). The first cell is non-visual. The latter two depolarize upon a light flash. The depolarization is largest for retinular cells because they transduce the light and send the signal through gap junctions to the eccentric cell, with some loss along the way. In the eccentric cell, action potentials fired by the axon are seen riding on the depolarization due to spike backpropagation into the soma. The amplitude of action potentials has been attenuated in the figure for better viewing of the depolarizing potential. The resting potential of the cells is -50mV.

## Discussion

We have illustrated how to perform ERG recordings, optic nerve recordings, and intraretinal recordings on horseshoe crabs in vivo. The recording techniques each provide different insights into the neural basis of vision, and they can all be used to study retinal function in live animals thanks to the crab's large eyes and hard carapace. Optic nerve activity can even be recorded from freely behaving animals in the ocean with proper construction of electrodes (4). These techniques can also be performed on excised eyes with minor modification of the setup. The relevance of such experiments to the natural condition would be limited, as the physiological properties of the crab's eye change when removed from the animal (5), but the instructional value would be great for a teaching lab given the widespread use of these methods in neuroscience.

ERG recording, optic nerve recording, and intraretinal recording were presented separately here for sake of clarity. In practice, multiple recording methods are often combined within a single experiment to simultaneously monitor changes in neural activity and visual sensitivity in one or both lateral eyes. The small chamber we use for ERG recording is particularly advantageous in this regard over other designs (1, 6). Moreover, it holds a reservoir of saline solution that is sealed from air, eliminating the stability problems associated with conventional wick electrodes that can dry out over time. The suite of possible vision experiments with Limulus is even greater than this because the same procedures described for nerve recording can be used for nerve stimulation by connecting the electrode leads to an electrical stimulator instead of a signal amplifier.
